# Complex Three-Dimensional Microscale Structures for Quantum Sensing Applications

**DOI:** 10.1021/acs.nanolett.3c02251

**Published:** 2023-10-09

**Authors:** Brian W. Blankenship, Zachary Jones, Naichen Zhao, Harpreet Singh, Adrisha Sarkar, Runxuan Li, Erin Suh, Alan Chen, Costas P. Grigoropoulos, Ashok Ajoy

**Affiliations:** † Laser Thermal Laboratory, Department of Mechanical Engineering, University of California, Berkeley, California 94720, United States; ‡ Department of Chemistry, University of California, Berkeley, California 94720, United States; § Advanced Biofuels and Bioproducts Process Development Unit, E. O. Lawrence Berkeley National Laboratory, Berkeley, California 94720, United States; ∥ Chemical Sciences Division, Lawrence Berkeley National Laboratory, Berkeley, California 94720, United States; ⊥ CIFAR Azrieli Global Scholars Program, 661 University Avenue, Toronto, ON M5G 1M1, Canada

**Keywords:** Quantum Sensing, 2-Photon Polymerization, ODMR, NV Center, Nanodiamond

## Abstract

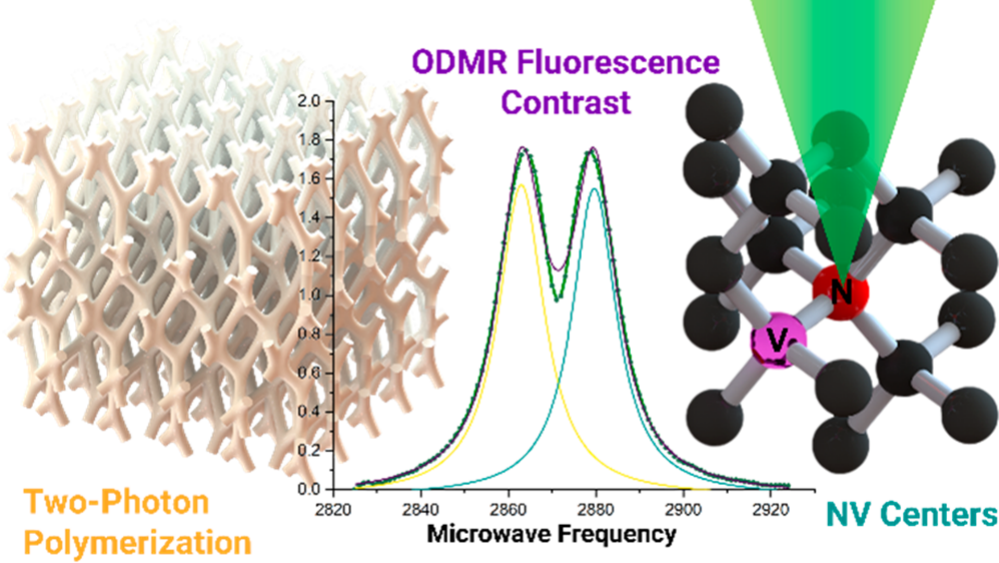

We present a novel method for fabricating highly customizable three-dimensional structures hosting quantum sensors based on nitrogen vacancy (NV) centers using two-photon polymerization. This approach overcomes challenges associated with structuring traditional single-crystal quantum sensing platforms and enables the creation of complex, fully three-dimensional, sensor assemblies with submicroscale resolutions (down to 400 nm) and large fields of view (>1 mm). By embedding NV center-containing nanoparticles in exemplary structures, we demonstrate high sensitivity optical sensing of temperature and magnetic fields at the microscale. Our work showcases the potential for integrating quantum sensors with advanced manufacturing techniques, facilitating the incorporation of sensors into existing microfluidic and electronic platforms, and opening new avenues for widespread utilization of quantum sensors in various applications.

Quantum sensing is an emerging technology that has enabled us to measure and observe the world around us at increasingly miniaturized scales.^1^ Quantum sensing approaches often use crystalline defects in wide-bandgap semiconductors, most notably nitrogen vacancy (NV) centers in diamond, based on their ability to host optically addressable spins with unique, spin-state selective photostable fluorescence and long spin coherence times.^2−5^ NV-based sensors can yield highly precise measurements of magnetic moments,^6−8^ electric fields,^9,10^ strain,^11^ and temperature^1,12,13^ with nanoscale spatial resolution. The design of sensors with useful architectures requires the combination of well controlled placement of NV centers as well as the ability to interrogate the quantum states of the sensors with high sensitivity. In practice the field has largely converged on two general approaches for deploying sensors, each with inherent limitations for structuring NV centers into useful configurations.^14^

One prevalent approach in the field involves modifying single crystalline diamond substrates through either etching processes, which create high surface area and high aspect ratio 2.5D features on the substrate’s surface,^15,16^ or directly implanting spin defects in 2D patterns near the surface of the substrate.^17−21^ However, these methodologies present challenges when attempting to create three-dimensional features like channels and overhangs.

Another approach utilizes nanodiamonds that contain high concentrations of NV centers, which are particularly attractive for intracellular sensing.^13,22^ Nonetheless, the techniques for fixing particles in space and creating designer sensor assemblies are still underdeveloped. Although intentional placement of nanodiamond particles can be achieved through optical trapping^8,23^ and the creation of permanent structures via complex particle self-assembly processes,^24,25^ these techniques are limited in terms of the design freedoms inherent to these processes.

Herein we demonstrate an approach to “3D-print” complex microscale polymeric structures with embedded nanodiamonds to achieve three-dimensional structuring of quantum sensors. Our goals are to demonstrate a fast, large-area fully 3D approach for arranging and positioning quantum sensors with submicrometer scale resolution, ultimately applying them in sensing applications.

We employ a two-photon polymerization (TPP) fabrication technique that provides new design flexibility for microscale applications. TPP is a 3D microfabrication process that utilizes high intensity, ultrashort laser pulses to initiate two photon absorption and the subsequent polymerization in photoresist.^26^ TPP can create three-dimensional structures hundreds of micrometers in size with feature resolutions below 100 nm,^27^ complex freeform surfaces,^28^ delicate overhangs,^29^ and freely moving, independent components.^26,30^ TPP is already widely employed for micro-optics,^28,31,32^ microfluidics,^33,34^ metamaterials,^35^ and emerging applications such as microneedles.^36^

Through two-photon lithography, we demonstrate the fabrication of arbitrarily shaped microstructures with diamond nanoparticles incorporated within them. Additionally, the microstructures can function as scaffolds for the placement of nanodiamond particles, enabling novel approaches to sensor arrangement. Quantum sensing of temperature and DC magnetic fields relies on optically detected magnetic resonance (ODMR) readout of the NV centers, accompanied by lock-in methods to mitigate autofluorescence from the structures and enhance sensitivity.

In the following we demonstrate the fabrication of complex overhanging structures, verifying the incorporation of NV centers into the structures and using a widefield imaging technique that can maximize optical contrast between NV center emission and the strong background photoluminescence from the TPP resin.

Structures are fabricated by employing sub-μm resolution direct femtosecond laser writing using two-photon polymerization (TPP) onto a biocompatible photoresist mixed with a concentrated solution of diamond nanoparticles. More details on the resin preparation and processing setup can be found in the Material and Methods section and in Supporting Information. Our TPP processing setup (Figure S1) is capable of high-speed laser writing up to 6 mm/s. We readily achieve feature resolutions down to 400 nm. Using stimulated emission depletion techniques like Wollhofen et al., we expect to be able to reduce feature sizes below 100 nm.^27^ Further discussions on print quality and comparison with the state of the art are elucidated in Table S1.

**Figure 1:**
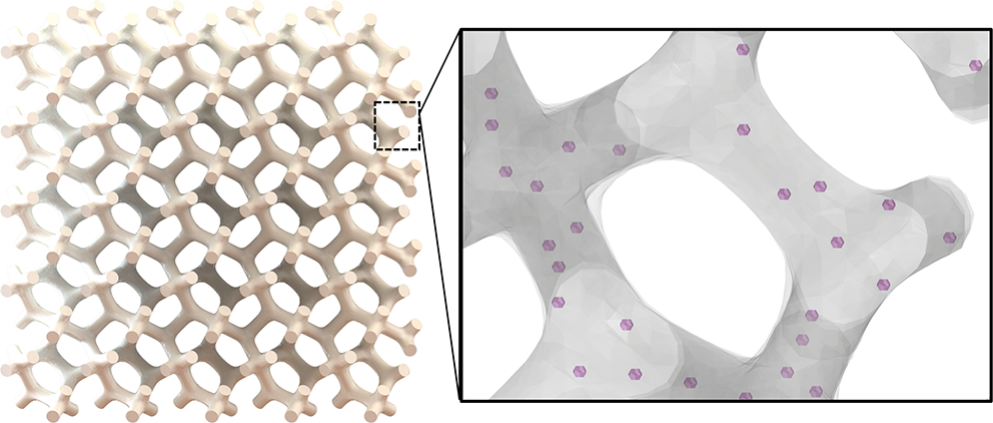
Motivation and strategy. (Left) Complex microscale porous structures as shown can enable new sensing capabilities but are impractical to fabricate with conventional lithography techniques. Two-photon polymerization is a technique well suited for fabricating complex 3D geometries with submicrometer resolution. (Right) By mixing in diamond particles into TPP compatible resins, we aim to fix particles into a permanent, arbitrarily shaped structure.

TPP is well suited for a variety of unmet applications in quantum sensing. For instance, TPP can construct complex porous structures and hollow microfluidic channels^37^ as well as mesoscale objects^38^ that can be patterned across large arrays without loss of feature resolution. Figure 2 displays representative structures fabricated with resin containing 25 and 100 nm nanodiamonds. Figure 2A,B exemplifies one such porous structure with a 2–5 μm pore size, suggesting potential applications in continuous-flow fluidic sensing. Given that TPP can be printed onto existing microfluidic chips, one could imagine using hybrid lithography processes to incorporate on-chip sensing elements for high-throughput assays.^39,40^

**Figure 2:**
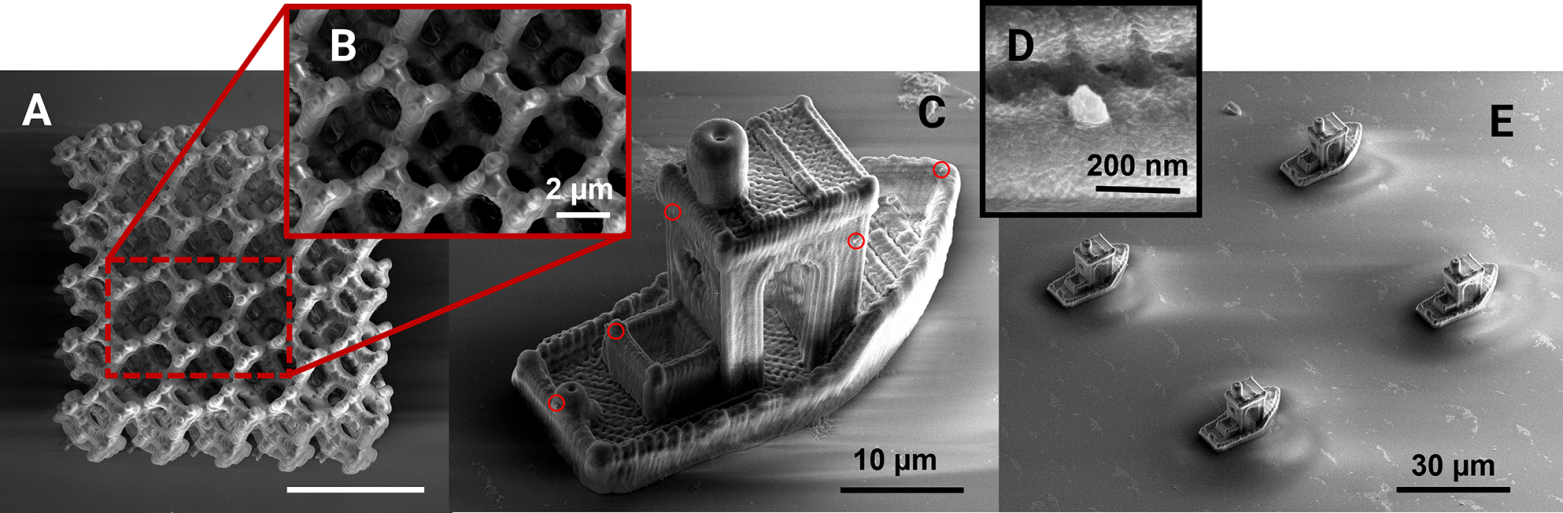
Example TPP structures. (A) SEM image of a porous tetrakaidecahedron structure exemplified in Figure 1 fabricated in our setup with resin containing 25 nm diameter diamond NV center particles (scale bar 25 μm) (B) with hollow interiors. (C) A microscale “Benchy” structure with submicrometer feature sizes patterned on a glass substrate (D) containing 100 nm diameter diamond particles where some are embedded onto the surface (circled) (E). These structures are arrayed to show the reproducibility and patterning capabilities of this technique with arrays of hundreds of elements being possible.

Figure 2C,E highlights the ability of TPP to generate finely detailed and complex geometries that can span hundreds or even thousands of micrometers. The depicted structure (“Benchy”) is incorporated with NDs and arrayed over a larger area. We estimate the volumetric fraction of diamonds in the structures after development to be ∼.2% based on ODMR images (see Figure 4C**)**. For 100 nm diameter particles this corresponds to ≈1 particle per 20 μm^3^, and for 25 nm particles we estimate there to be approximately ≈1 particle per μm^3^. These ratios are largely tunable based on the relative concentration of diamond solution to resin. Inevitably, given the high concentration of diamonds, several diamonds visibly appear on the surface of the structures in Figure 2. These are highlighted as circles in Figure 2C. Figure 2D depicts a close-up view of one of these 100 nm diamond particles lodged onto the surface of the parent structure. From the demonstrations in Figure 2, it is conceivable to fabricate micro lens arrays or photonic elements embedded with quantum sensors, enabling large field-of-view sensing applications.

Quantum sensing measurements utilizing ODMR rely on the spin-state-dependent fluorescence of NV center defects. Figure 3A presents a simplified model of an NV center in diamond, depicting it as a two-electron system with energy levels illustrated in Figure 3B. The ground triplet spin state exhibits zero field splitting (ZFS) at 2.87 GHz. The Hamiltonian of this system is described in Supporting Information.

**Figure 3:**
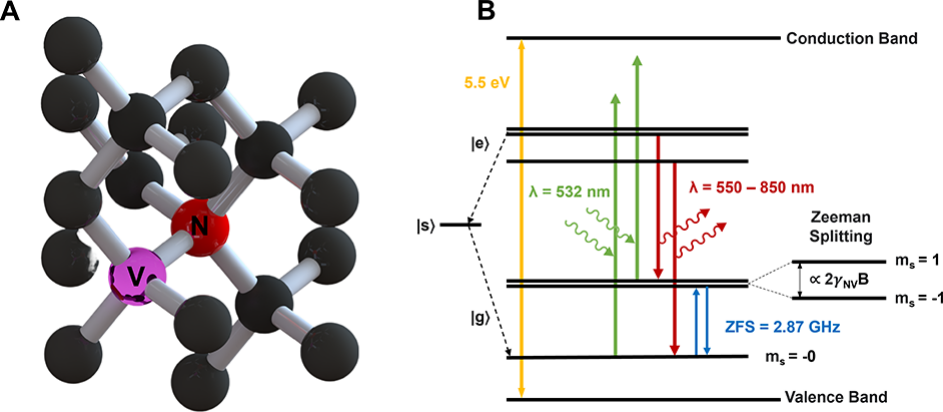
Diamond NV center structure and energy level diagram. (A) A nitrogen defect (red) is implanted adjacent to a vacant lattice site within a carbon bulk. In typical diamond nanoparticles, NV defects are present at concentrations ranging from 1 to 5 ppm (ppm). (B) The fluorescence and spin polarization of the NV center depend on its spin relaxation dynamics. Upon optical excitation (green arrows), the nonradiative intersystem crossing to the singlet state exhibits a higher probability when excited to the *m*_s_= +1 spin sublevel compared to the *m*_s_ = 0 sublevel. This disparity enables the measurement of spin polarization through the fluorescence intensity.

When microwaves (MWs) are applied resonant with the transitions between spin sublevels, the optical emission intensity undergoes a change, providing a means to optically probe the energy levels through a sweep of MW frequency. In the presence of an external magnetic field, the degeneracy of the |*m*_S_ = ±1⟩ magnetic spin sublevels is lifted, while the ZFS value itself is temperature-dependent. These ODMR measurements can therefore be employed for tasks such as magnetic field or temperature sensing.

To understand the emissive characteristics of our functionalized TPP structures, we first measure the photoluminescence spectra of both the NV center containing nanodiamonds and the postprocessed photoresist individually. The emission spectra under 532 nm excitation of the two materials (Figure 4A) show broad overlap between ∼575 and 675 nm. Despite filtering our fluorescence readout to wavelengths of >680 nm the optical signal is orders of magnitude larger than that of the NV centers based on the compositional fraction of the two species. To isolate the weaker emission of the NV centers from the background resin, we utilize a custom microscope (Figure S2A) capable of applying amplitude modulated microwaves across the NV center spin state transition frequencies range of interest (∼2.800–2.925 GHz). Since only the NV center fluorescence intensity is modulated by the application of the microwaves within this range, the oscillating signal is extracted and amplified with a lock-in amplifier.

**Figure 4:**
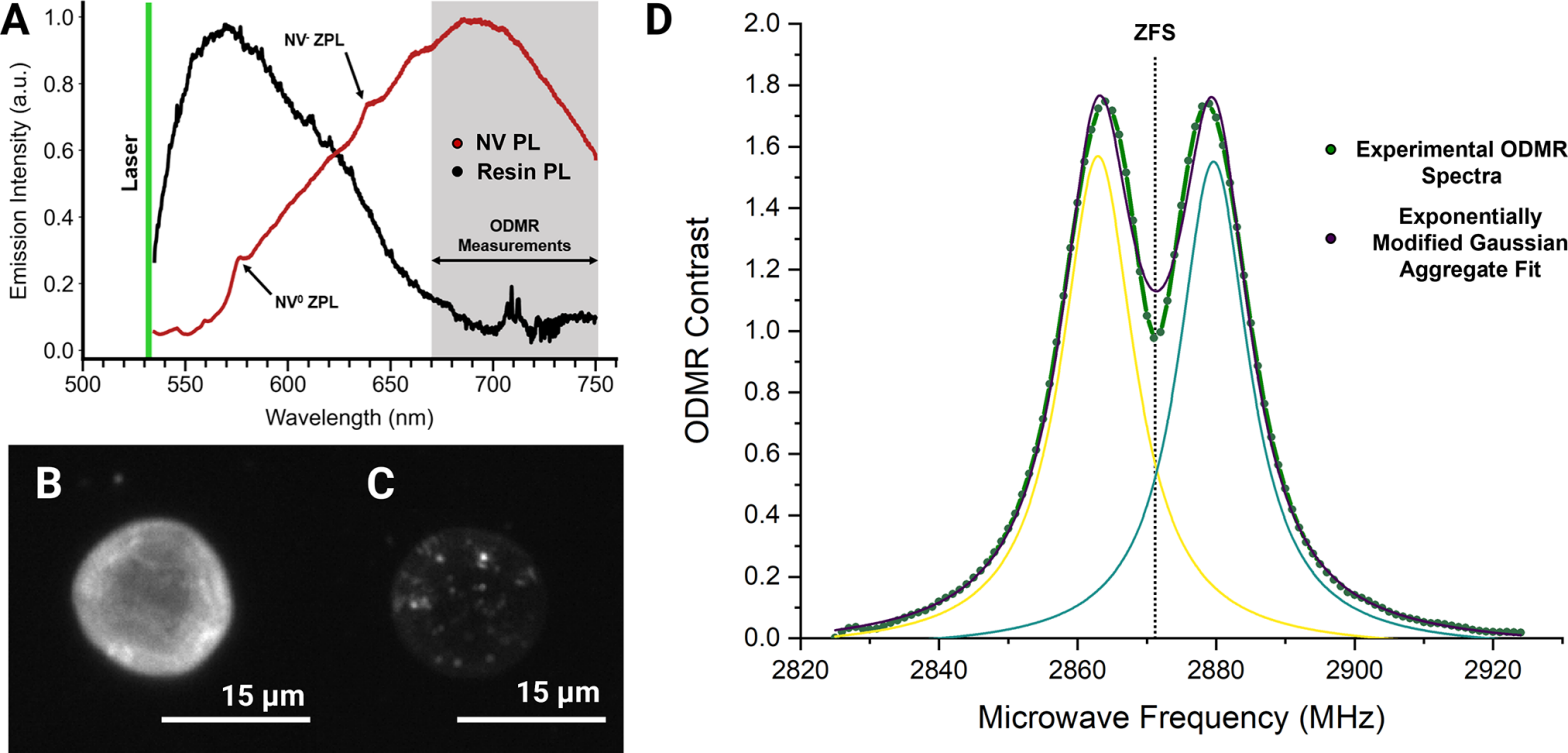
ODMR imaging. (A) Photoluminescent spectra of NV centers and SZ2080 resin as the TPP matrix. (B) Fluorescence image of a cylindrical TPP structure (C) in comparison to an ODMR fluorescent contrast image taken at 2870 MHz which clearly discerns the relative spatial location of NV center containing diamond nanoparticles. (D) ODMR contrast spectra, *I*′_contrast_, of a pillar structure taken in ambient conditions.

Initially, to verify the successful incorporation of diamond nanoparticles into our TPP structures, we construct a widefield ODMR contrast image of a 15 μm diameter, 5 μm tall cylindrical structure, containing 100 nm diamond particles by applying microwaves at 2.87 GHz and subtracting out the background fluorescence (Figure 4C). This methodology is employed in a variety of other works on NV center sensing.^22,41−44^ In this image, the intensity of each camera pixel is determined by
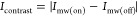
1

In these fluorescent contrast images (Figure 4C) we can clearly discern and locate at least 30 individual and possibly aggregated diamond particles within their fluorescent polymer matrix whereas these distinctions cannot be made in the corresponding fluorescent image of the pillars (Figure 4B). By using a more sensitive multipixel photon counter (MPPC) and sweeping across a range of microwave frequencies following the pulse sequence shown in Figure S2B, we construct the ODMR contrast spectrum shown in Figure 4D wherein percent contrast is defined as
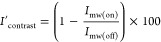
2

Subsequently we fit two exponentially modified Gaussian functions to the data. At ambient conditions, we observe maximum ODMR contrasts at ∼2.8616 and ∼2.8792 GHz. This low-field splitting is a remnant of residual strain in the diamond lattice after the fabrication of nanoparticles. Using our NV-pillar structures, we achieve a maximum ODMR contrast in the range of 1.2–2.5% for different structures.

When varying the temperature of the diamond lattice in the NV molecular model theorized by Doherty et al.,^45^ the splitting parameter *D* in the zero-field splitting term can be expressed as
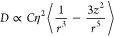
3where *C* is the spin–spin interaction constant, η the electron density, and  the interaction of sp^3^ electron densities in carbon atoms. As the temperature of the diamond nanoparticle increases, we expect thermal expansion of the lattice that increases the lattice spacing between atoms, *r*, resulting in a decrease in the splitting parameter.^45^ In the ODMR spectra, this manifests as a shift in the zero-field splitting to lower frequencies. Within relatively small ranges of temperature, the change in the splitting parameter is locally linear.

In our experiments, we investigate optical thermometry of 5 μm tall, 15 μm diameter cylindrical structures in temperature ranges varying from 295K to 323 K which are relevant to biological applications. Temperature is varied in these experiments by using a heating stage, which is allowed to reach thermal equilibrium before collecting each temperature measurement. The resultant ODMR spectra are shown in Figure 5A. From these spectra, we can calculate the ZFS and uncertainty by fitting a two-peak exponentially modified Gaussian function to each spectrum, the results of which are shown in Figure 5B. We observe a strong linear relationship of −0.07447 ± 0.00154 MHz/K which agrees with measurements from Doherty et al.^45^ and achieve an average temperature sensitivity of 0.645 K/ Details on the sensitivity calculations are provided in the Supporting Information.

**Figure 5:**
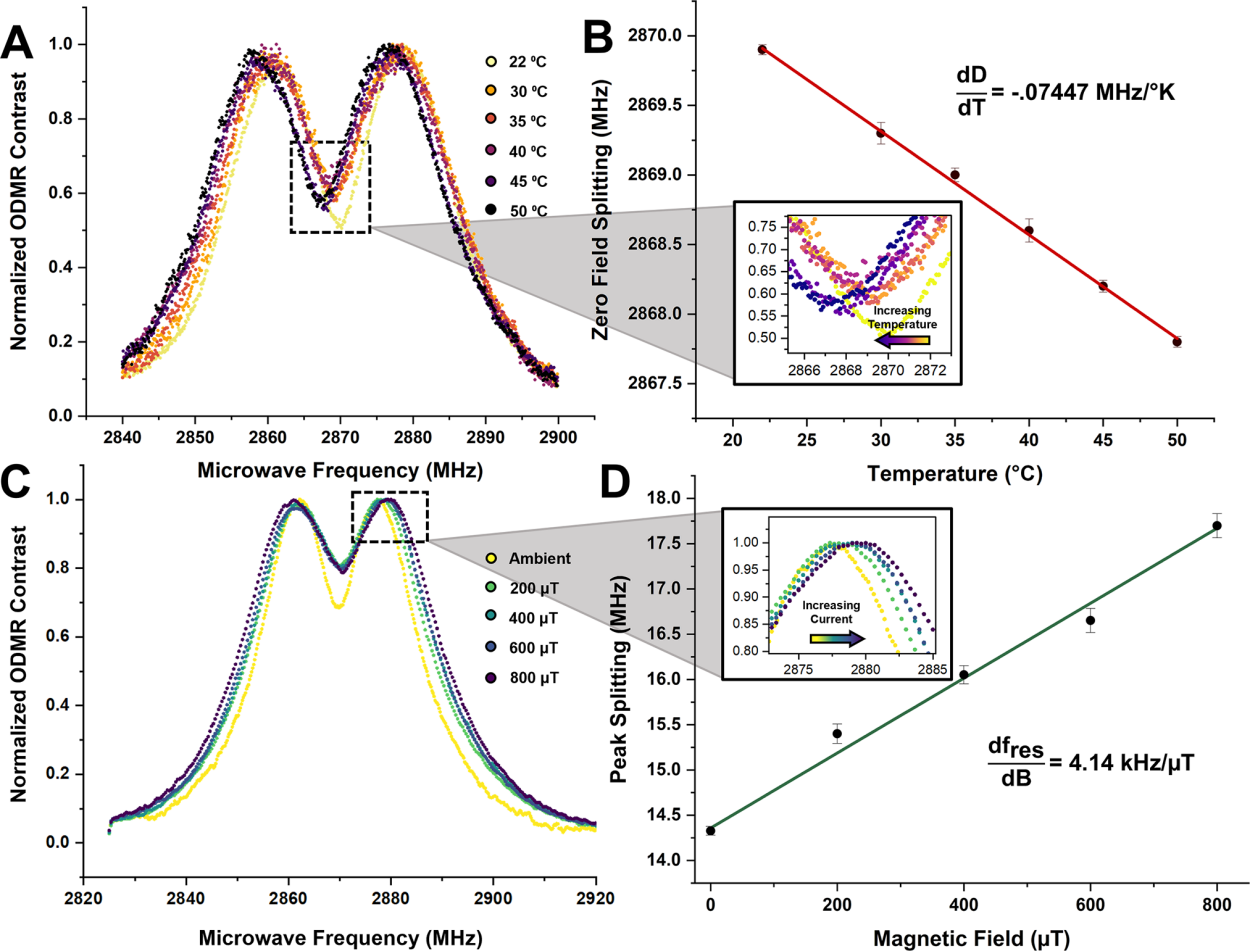
Quantum sensing measurements. (A) ODMR contrast spectra of NV center containing microstructures normalized to maximum contrast at varying temperatures (22–50 °C) relevant to biothermometric applications. (B) Zero-field splitting in ODMR as a function of changing temperature where a clear locally linear relationship of −.07447 MHz/K is observed. Error bars are 95% confidence intervals. (C) ODMR contrast spectra of sensing structures at varying applied magnetic fields from a nearby thin wire normalized to maximum contrast. (D) Splitting of *m*_s_ = ±1 peaks as a function of applied current in which we see the splitting of the two constituent curves increase with increasing field at 4.14 kHz/μT. Error bars are 95% confidence intervals.

Lock-in ODMR techniques excel at improving the sensitivity of temperature measurements and the structuring enabled by TPP provides capabilities that are not achievable for other methods of NV based thermometry. For example, in prototypical biological applications NV centers are disbursed into solution and local temperature measurements of cells and organisms are dependent on intercellular uptake of particles. Due to diffusion in the cellular fluid medium, sensing particles would not maintain a set distance from the observables of interest. Incorporating the particles into scaffold members could provide extracellular support structure onto which the cells attach that simultaneously acts as a medium for temperature and magnetic field sensing. More traditional ODMR techniques likewise suffer from the background autofluorescence of their surroundings, limiting the ability to extract measurement signals in complex environments. Since only the NV centers fluorescence is modulated within the range of relevant microwave frequencies, we can effectively isolate the ODMR signal and measure a maximum observed contrast signal to >2% with high signal-to noise ratio which is on par with measurements observed elsewhere in the literature.^7^

In an ensemble of nanodiamonds, the quantization axis for the NV centers, aligned with the N-to-V lattice axis, is randomly oriented. Consequently, NV centers within each particle encounter a distinct magnetic field projection along this direction compared to NV centers in other particles within the resin matrix. In the collected ODMR spectra, this is manifested as an increase in the splitting of the two peaks as well as a broadening of the individual peaks. This broadening is explained in greater detail in Figure S3.

We perform a demonstrative experiment by passing a variable current through a thin wire placed roughly 50 μm away from a stationary cylindrical structure. We collect ODMR measurements at the same distance from the wire for each current. We estimate the magnetic field by using an infinite wire approximation. The results of this experiment are shown In Figure 5C, where discernible splitting effects are observed. By fitting exponentially modified Gaussian functions to these data, we calculate the splitting between peaks of the constituent curves as well as an associated uncertainty and plot the results in Figure 5D. A linear fit yields a relationship of 4.14 ± 0.209 kHz/μT for this experimental system. The splitting parameter is approximately an order of magnitude smaller than what is typically observed in NV centers (28 kHz/μT) and is a result of the off-axis projection of the magnetization axis onto the randomly oriented particles. We find an average sensitivity of approximately 216.4 μT/ using a similar estimation procedure as in the temperature measurements. We anticipate that more advanced protocols, e.g., employing a Ramsey sequences, can be employed to yield better measurement sensitivity.^46,47^

The ability to incorporate diamond particles containing NV centers into our TPP resin gives extraordinary design freedoms for building three dimensional structures with microscale features that can be patterned or arrayed across a substrate or device. This simultaneously enables sensing applications for point mapping across large areas as well as 3D mapping of microscale volumes. Herein we have demonstrated the feasibility of measuring ODMR spectra within single structures by using lock-in amplification to isolate NV center emission from the fluorescent background. We leave room for further work to apply these techniques to demonstrate large field 2D and microscale 3D imaging for application specific experiments.

TPP has already found use in a number of applications discussed previously. Embedding nanodiamonds into structures for these applications would allow for enhanced diagnostic capabilities that could add new dimensionalities to the possible *in situ* measurements of these systems. We can also envision a number of new applications that are discussed in the Supporting Information including functionalized cellular scaffolding, remote detection of passing currents on microelectronics, and chemical sensors in microfluidics.

While this work focuses on optical detection of magnetic resonance of NV center particles, it should be noted that spin active defects occur in other systems such as silicon vacancy SiC, hBN, and silicon at mostly cryogenic temperatures.^3,5,48^ Similar techniques for incorporating color center particles into TPP resins could allow for tunable sensing properties.

We have demonstrated a technique for developing “designer” microscale 3D structures relevant for quantum sensing by mixing nanoparticles containing NV centers into TPP compatible resins. We observe that autofluorescence from the bulk resin matrix is more than an order of magnitude larger than the ODMR signal and thus construct a differential contrast microscope using lock-in amplification at the applied microwave amplitude pulse frequency to isolate the ODMR signal. Finally, we show that combining these two techniques allows us to measure temperature and magnetic field measurements. Further work should focus on the development of analogous 3D imaging techniques using confocal microscopy and exploring practical end applications where this technique would be advantageous.

## Materials and Methods

A hybrid organic–inorganic resin, SZ2080 is used with Zr-DMAEMA (30 wt %) as a binder. The resin is composed of 70 wt % zirconium propoxide and 10 wt % (2-dimethylaminoethyl) methacrylate (DMAEMA) (Sigma-Aldrich). A suspension of either 25 (∼1 ppm of NV) or 100 nm (∼3 ppm of NV) diameter NV center diamond particles in water (Adamas Nanotechnologies) was included in the resin at a ratio of 1:50 (v/v) and mixed for 30 min. Diamonds were fabricated via HPHT synthesis with ∼100 ppm of substitutional nitrogen. Diamonds are then irradiated with 2–3 MeV electron radiation and subsequently annealed.

Structures were fabricated by submicrometer resolution direct femtosecond laser writing using two-photon polymerization on SZ2080 photoresist. The source laser is a FemtoFiber Pro NIR laser, which emits 780 nm, 100 fs fwhm, pulses at 80 MHz. Polymerization of the resin is achieved with a 40× microscope objective lens (Plan-Apochromat 40×/1.3 Oil Olympus). The laser output energy was measured before the objective lens at 6.6 mW, and the scanning speed of the laser spot at the imaging plane was set to 1000 μm/s and is steered by using a two-axis optical galvanometer. The resin sample is positioned with three axis piezo and servo stages. Structures are built by building successive layers in the optical axis by scanning the laser across the imaging plane at set heights.

Photoluminescence measurements were taken with a Renishaw InVia spectrometer with an 1800 lines/mm grating.

ODMR is measured using a custom-built wide-field fluorescence microscope discussed in detail in Supporting Information. A diagram of the microscope is shown in Figure S2.

## Supporting Information Available

Two-photon polymerization setup, print quality comparison to literature, ODMR microscope and pulse sequence, fitting functions, NV center Hamiltonian, sensitivity calculations, magnetic field peak broadening simulations, and table on potential applications (PDF)
