# Supplementary material for: Complex Three-Dimensional Microscale Structures for Quantum Sensing Applications
Source: Nano Lett. 2023 Oct 9;23(20):9272–9. doi: 10.1021/acs.nanolett.3c02251 (PMC10603797; doi:10.1021/acs.nanolett.3c02251)
Supplement: Supplementary file 1 — nl3c02251_si_001.pdf [file nl3c02251_si_001.pdf]

Supporting Information for: Complex 3-Dimensional Microscale Structures for Quantum Sensing Applications

Brian W. Blankenship[1,2†,] Zachary Jones[2,3†], Naichen Zhao[1], Harpreet Singh[2], Adrisha Sarkar[2], Runxuan Li[1], Erin Suh[1], Alan Chen[1], Costas Grigoropoulos[1*], Ashok Ajoy[2,4*]

1. Laser Thermal Laboratory, Department of Mechanical Engineering, University of California, Berkeley, CA 94720, USA
2. Department of Chemistry, University of California, Berkeley, CA 94720, USA
3. Advanced Biofuels and Bioproducts Process Development Unit, E. O. Lawrence Berkeley National Laboratory, Berkeley, CA 94720, USA
4. Chemical Sciences Division, Lawrence Berkeley National Laboratory, Berkeley, CA 94720, USA

† These Authors contributed equally
*Corresponding Author

Email: ashokaj@berkeley.edu, cgrigoro@berkeley.edu

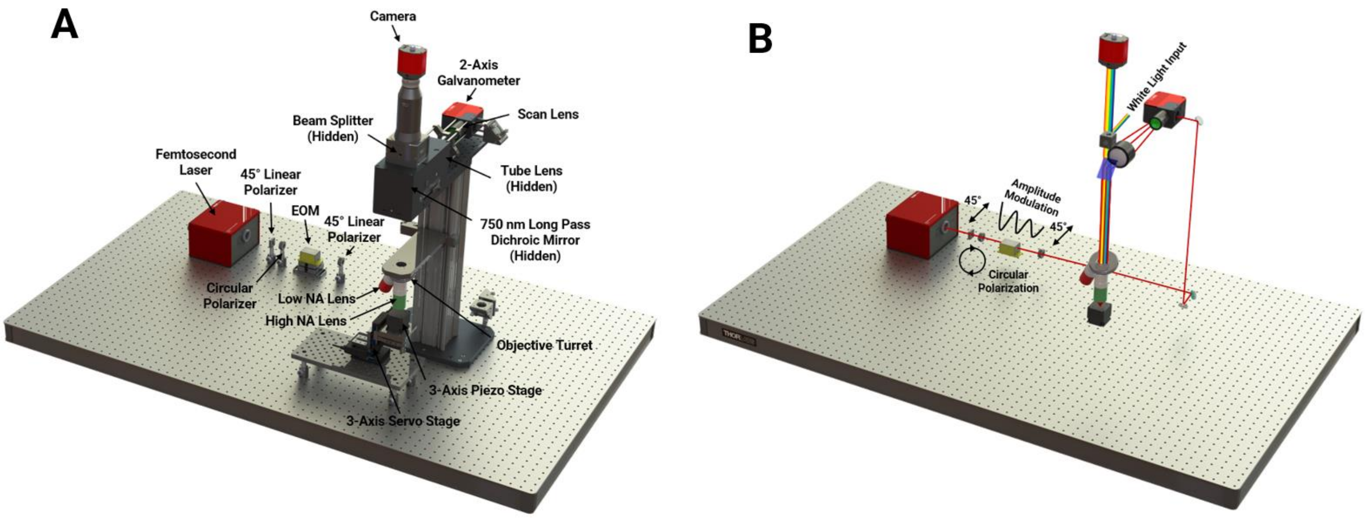

Figure S1: Two-Photon Polymerization Setup (A) Rendering of the TPP processing setup with adjustable power modulation and laser scanning capabilities. B) A circularly polarized femtosecond laser beams phase is modulated by an EOM and linear polarizer at the output. This beam is scanned by a galvanometer for fast (> 1000 µm/s) scanning at the image plane. A beam splitter allows a separate imaging light to image while printing.

Table S1: TPP Print Quality and NV Composition in Resin

In the present work we build structures using a custom TPP setup capable of achieving 500nm feature sizes with a 1.3 NA objective at laser powers and galvanometer speeds specified in the paper. Using state of the art techniques such as stimulated emission depletion lithography (STED), TPP can construct objects with sub-55 nanometer feature resolution. Varying galvanometer speeds allows us to achieve print rates in the range of 2,000 - 10,000 μm^3/s whereas state of the art projection-type printers can reach print rates approaching 1,000,000 μm^3/s with micron-level resolution.

We estimate that the density of particles in our TPP structures is approximately 1 particle per 20 μm^3. This is controllable to some degree based on the ratio of diamonds to resin used in the printing medium. For our sensing measurements we utilize 100nm diameter NV center particles with 3 ppm NV centers from Adamas Nanotechnologies and estimate there to be 2200 NV centers per diamond particle.

	Minimum feature resolution	Volume printed per second	Maximum geometry constraints	Estimated particles per unit volume	NV centers per particle
Present Work	~400 nm	2,000 - 10,000 μm^3/s	200 µm (xy)<br>170 µm (z)	~.05 μm^{-3}	~2200
Current state of the art	< 55 nm[1,2]	~1,000,000 μm^3/s	>1mm (xy)[3]<br>> 450 µm (z)[4]	N/A	N/A

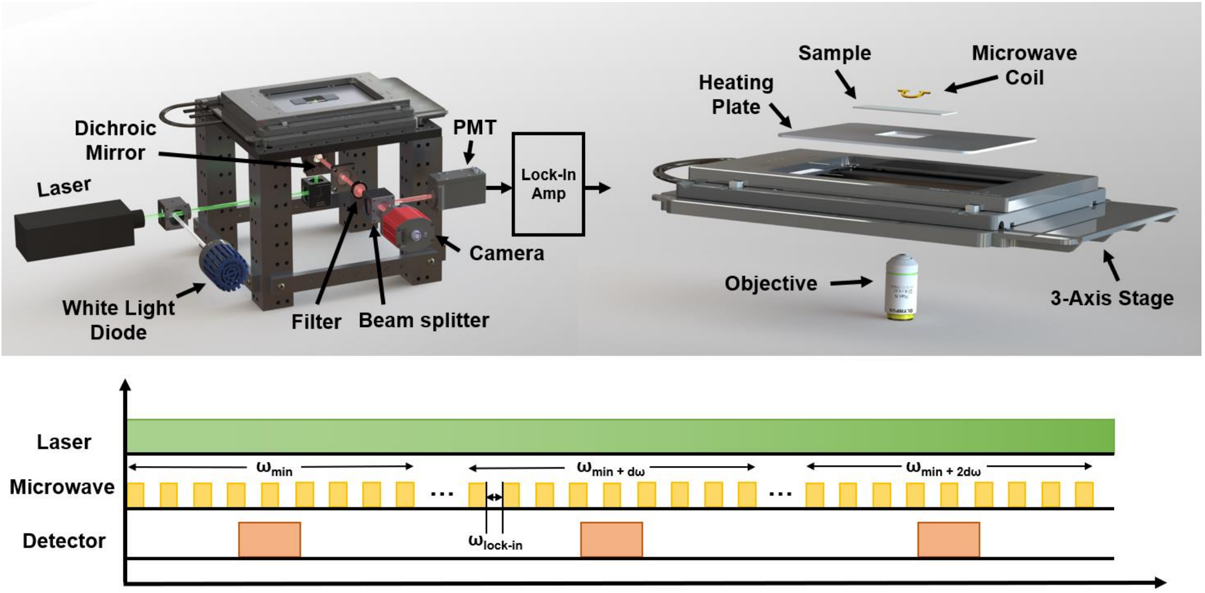

Figure S2: ODMR Microscope and Pulse Sequence (A) Left: Rendering of the ODMR microscope and beam path. A 532 nm excitation laser (Coherent Verdi G) and white light imaging source (CoolLED PE-4000) are directed to an objective (Olympus LMPLFLN 20X) via a dichroic mirror (Thorlabs FEL0600-1) and focused onto the sample. Lower energy photons from sample emission pass through the 600 nm longpass dichroic mirror and a 680 nm bandpass filter (FF01-680/42-25) to a tube lens. A beamsplitter directs approximately 30% of the light to an imaging sCMOS camera (Teledyne Kinetix) for imaging and 70% to a multi-pixel photon counter (Hamamatsu C14455) for ODMR measurements. The MPPC signal is amplified using a lock-in amplifier (SRS SR830) with reference frequency set to the microwave pulse frequency. Microwave excitation is generated using a synthesizer (HP 8664A) and 100% amplitude modulated using a switch (minicircuits ZYSW-2-50DR, Empower 1146-BBM4A6AK5). This modulating signal is then amplified by a series of amplifiers (minicircuits ZYSW-2-50DR) to a final power of approximately 50 W) Right: Closeup of the stage. A heating plate controls the temperature of the sample while a small microwave coil applies pulsed microwaves. (B) ODMR pulse sequence. A 100mW CW laser continuously excites the sample while a variable frequency microwave at constant pulse frequency irradiates the sample. The MPPC captures signal during both "on" and "off" phases of the microwave pulse.

Exponentially Modified Gaussian Function Fitting

Our data is fit to exponentially modified gaussian functions of form:

$$f(x;\ \mu,\sigma,\lambda) = \frac{\lambda}{2} e^{\frac{\lambda}{2}(2\mu+\lambda\sigma^2-2x)} erfc\left(\frac{\mu+\lambda\sigma^2-x}{\sqrt{2}\sigma}\right)$$

Where erfc is the complementary error function defined as:

$$erfc(x) = 1 - erf(x) = \frac{2}{\sqrt{\pi}}\int_x^{\infty} e^{-t^2} dt$$

The exponentially modified gaussian function is the convolution of the normal and exponential probability density functions and was found to be the best fit for our ODMR contrast data.

NV Center Hamiltonian

The Hamiltonian of an NV center system can be described by:

$$H_{NV} = D\left({S_z}^2 - \frac{1}{3}S^2\right) + \gamma_e B_0(S_x \sin\theta + \ S_z \cos\theta) + SAI$$

Where D is the zero-field splitting (ZFS) parameter, $\gamma_e = 28\ MHz/mT$ is the electron gyromagnetic ratio, S represents spin angular momentum, B is the magnetic field, A the hyperfine tensor, I the spin operator of the nitrogen nucleus and θ is the angle between the magnetic field and NV axis. By precisely measuring the spectral emissions governed by the above Hamiltonian we measure the local temperature and magnetic field in our structures.

Changes in the measured ODMR spectrum can be used as a proxy to measure the local conditions of the ensemble of NV centers inside each structure. The Hamiltonian in equation (1) can be understood as the sum of the zero-field splitting, electron Zeeman term, and the hyperfine coupling:

$$H = \ H_{ZFS} + H_{Zeeman} + \ H_{HF}$$

Where differences in temperature more strongly affect the contribution of the zero-field splitting term in the Hamiltonian, and the Zeeman term is largely influenced by the magnetic field effectively allowing a means to decouple measurements of magnetic field and temperature.

Sensitivity Calculations

Sensitivity was calculated in the following way:

$$\text{Sensitivity } \eta = \frac{\sigma_P\sqrt{2\delta t_{int}}}{\frac{dX}{dY}}$$

Where σ_P is the standard error of a point measurement in units of MHz, δt_{int} is the integration time of the point, and $\frac{dX}{dY}$ is the change in position X in units of MHz with respect to parameter Y (temperature, magnetic field).

Figure S3: Magnetic Field Broadening Simulations

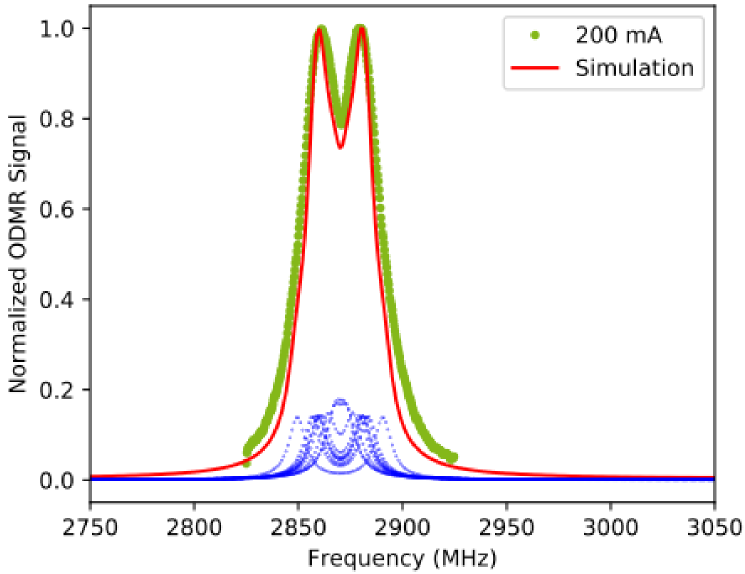

We performed simulations of an ensemble of randomly oriented NV centers in order to understand why we observed broadening of peaks in the presence of magnetic fields. In the graph, the red curve is the sum of the ODMR signal of ten randomly oriented NV centers simulated with the Lindblad equation. The Lindblad equation is integrated for half a microsecond, and the ODMR signal is calculated from the resulting density matrix. The Range-Kutta method is used for numerical integration. A similar methodology is employed in Singh et al.[5]

Table S2: Outlook of TPP Quantum Sensing

Field	Application	Challenges	Solutions
**Biology**	2/3D Bio-Thermometry 3D Bio-Thermometry	Diffusion of particles in-vivo prevents imaging of NV centers at a fixed location.	Construction of 2D cellular scaffolds (see Trautmen et. al.[6]) with nanodiamonds integrated into the resin matrix for fixed-position imaging.
	Neuronal Imaging	Photobleaching of fluorophores over prolonged exposures.	Construction of 2D/3D cellular scaffolds (See Accardo et. al.[7]) with nanodiamonds integrated into the resin matrix for fixed-position imaging. NV centers are also not photobleachable.
**Semiconductors**	Remote Detection of Passing Currents	Commercial magnetometers cannot be used for sub-millimeter detection volumes and NV centers cannot ordinarily be patterned onto non-diamond substrates.	Structures containing NV centers can be patterned onto most substrates and can be localized to microscale detection domains.
	Widefield Localized Surface Temperature Measurements	Surface thermometry techniques such as TDTR require addition of thin films onto substrate, thermocouples are bulky and require wired connections.	Structures containing NV centers can be printed onto most substrates, can be localized to microscale detection domains, requires no wires, and measurements are independent of optical properties of substrates/thin films.
**Microfluidics**	Chemical Sensing	Microfluidic channels are printed onto costly diamond substrates.	Porous structures can be directly printed into microfluidic cavities with sub 60 nm resolution feature sizes for micro/nanoliter confined volume NMR.
	Flow Temperature Measurements	Microfluidic channels must be printed onto costly diamond substrates or incorporate diamond particles into flow. Anemometers dependent on fluid/flow properties.	Fixed structures along a microfluidic channel can be constructed to monitor temperature at fixed locations. Measurements are independent of fluid/flow properties.

References

(1) Wollhofen, R.; Katzmann, J.; Hrelescu, C.; Jacak, J.; Klar, T. A. 120 Nm Resolution and 55 Nm Structure Size in STED-Lithography. *Opt. Express* **2013**, *21* (9), 10831–10840. https://doi.org/10.1364/OE.21.010831.

(2) Gan, Z.; Cao, Y.; Evans, R. A.; Gu, M. Three-Dimensional Deep Sub-Diffraction Optical Beam Lithography with 9 Nm Feature Size. *Nat. Commun.* **2013**, *4* (1), 2061. https://doi.org/10.1038/ncomms3061.

(3) Jonušauskas, L.; Gailevičius, D.; Rekštytė, S.; Baldacchini, T.; Juodkazis, S.; Malinauskas, M. Mesoscale Laser 3D Printing. *Opt. Express* **2019**, *27* (11), 15205–15221. https://doi.org/10.1364/OE.27.015205.

(4) Obata, K.; El-Tamer, A.; Koch, L.; Hinze, U.; Chichkov, B. N. High-Aspect 3D Two-Photon Polymerization Structuring with Widened Objective Working Range (WOW-2PP). *Light Sci. Appl.* **2013**, *2* (12), e116–e116. https://doi.org/10.1038/lsa.2013.72.

(5) Singh, H.; Hollberg, M. A.; Anisimov, A. N.; Baranov, P. G.; Suter, D. Multi-Photon Multi-Quantum Transitions in the Spin-$\frac{3}{2}$ Silicon-Vacancy Centers of SiC. *Phys. Rev. Res.* **2022**, *4* (2), 023022. https://doi.org/10.1103/PhysRevResearch.4.023022.

(6) Trautmann, A.; Rüth, M.; Lemke, H.-D.; Walther, T.; Hellmann, R. Two-Photon Polymerization Based Large Scaffolds for Adhesion and Proliferation Studies of Human Primary Fibroblasts. *Opt. Laser Technol.* **2018**, *106*, 474–480. https://doi.org/10.1016/j.optlastec.2018.05.008.

(7) Accardo, A.; Blatché, M.-C.; Courson, R.; Loubinoux, I.; Vieu, C.; Malaquin, L. Two-Photon Lithography and Microscopy of 3D Hydrogel Scaffolds for Neuronal Cell Growth. *Biomed. Phys. Eng. Express* **2018**, *4* (2), 027009. https://doi.org/10.1088/2057-1976/aaab93.
